# Role of ferroptosis-associated genes in ankylosing spondylitis and immune cell infiltration

**DOI:** 10.3389/fgene.2022.948290

**Published:** 2022-11-11

**Authors:** Qiaochu Li, Zhiyu Chen, Chaohua Yang, Linbang Wang, Jingjin Ma, Tao He, Huanhuan Li, Zhengxue Quan

**Affiliations:** ^1^ The First Clinical College, Chongqing Medical University, Chongqing, China; ^2^ Department of Orthopedics, The First Affiliated Hospital of Chongqing Medical University, Chongqing, China; ^3^ Department of Orthopaedic Trauma, Chongqing General Hospital, Chongqing, China

**Keywords:** ankylosing spondylitis, ferroptosis, immunity, DDIT3, HSPB1

## Abstract

Ankylosing spondylitis (AS) is a chronic progressive autoimmune disease with insidious onset, high rates of disability among patients, unknown pathogenesis, and no effective treatment. Ferroptosis is a novel type of regulated cell death that is associated with various cancers and diseases. However, its relation to AS is not clear. In the present study, we identified two potential therapeutic targets for AS based on genes associated with ferroptosis and explored their association with immune cells and immune cell infiltration (ICI). We studied gene expression profiles of two cohorts of patients with AS (GSE25101 and GSE41038) derived from the gene expression omnibus database, and ferroptosis-associated genes (FRGs) were obtained from the FerrDb database. LASSO regression analysis was performed to build predictive models for AS based on FRGs, and the ferroptosis level in each sample was assessed *via* single-sample gene set enrichment analysis. Weighted gene co-expression network and protein-protein interaction network analyses were performed for screening; two key genes, DDIT3 and HSPB1, were identified in patients with AS. The relationship between key genes and ICI levels was assessed using the CIBERSORT algorithm, followed by gene ontology and Kyoto Encyclopedia of Genes and Genomes pathway enrichment analyses. Finally, DDIT3 and HSPB1 were identified as diagnostic markers and potential therapeutic targets for AS. DDIT3 was highly positively correlated with the infiltration levels of various immune cells, while HSPB1 was negatively correlated with the infiltration levels of several different types of immune cells. In conclusion, DDIT3 and HSPB1 may induce ferroptosis in the cells of patients with AS *via* changes in the inflammatory response in the immune microenvironment, and these genes could serve as molecular targets for AS therapy.

## Introduction

Ankylosing spondylitis (AS) is a chronic progressive autoimmune disease characterized by inflammation of the sacroiliac joints and spinal entheses. It is often observed in males below 40 years of age, with an overall incidence of approximately 0.5% in the United States (Dean et al., 2014). AS has an insidious onset and may show no symptoms or only non-specific clinical symptoms, such as weakness, wasting, and mild anemia during the early stages. Due to mild symptoms, patients may fail to notice the disease in its early stages, which may result in disease progression and missing the best treatment window. In the late stages of AS, the deformity and limited motion of spinal segments and joints, the appearance of extra-articular lesions, as well as pathological changes in the gastrointestinal tract, heart, eyes, and nervous system seriously affect the quality of life of patients (Landewé et al., 2009; Maksymowych, 2010; El Maghraoui, 2011; Dean et al., 2014; Fallahi and Jamshidi, 2016). Common treatments for AS include the administration of non-steroidal anti-inflammatory drugs to reduce or relieve inflammation and physical/sports therapy to maintain normal posture, realize the optimal functional position of the body, and prevent deformity. Patients with severe symptoms may require surgical interventions (Zochling, 2008; Pécourneau et al., 2018; Ritchlin and Adamopoulos, 2021). The prognosis of AS varies significantly among individuals. It would be difficult to cure the disease without having a complete understanding of its pathogenesis, and the progression of the disease can be slowed only with targeted treatment. Thus, a deeper understanding of the AS pathogenesis is crucial. Although AS is highly inheritable, the details of its pathogenesis have not yet been elucidated. Since its discovery in 1973, HLA-B27 has been considered the primary genetic factor influencing the development of AS. Nevertheless, the polygenic nature of AS has been demonstrated, and new molecular pathways are being discovered gradually over time, leading to further understanding of the pathogenesis of AS (Brown and Wordsworth, 2017; Ranganathan et al., 2017).

Cell death is an event that occurs throughout the lifetime regardless of physiological or pathological triggers. The balance between cell death and survival is essential for normal development and *in vivo* homeostasis. Cell death is also a diverse process exhibiting variable characteristics depending on the altered physio-pathological environment (Hotchkiss et al., 2009). The Nomenclature Committee on Cell Death has characterized various methods of cell death, including apoptosis, necrosis, pyroptosis, autophagy, and ferroptosis, based on cell morphology and biochemical and functional features (Kroemer et al., 2009). Non-apoptotic types of cell death may help selectively eliminate and thereby prevent the activation of potential “malignant” cells in certain pathological states. Ferroptosis is a novel mechanism of regulated cell death (RCD). Induction of cell death in ferroptosis is dependent on the overload of free ferrous ions and catalysis of lipid peroxidation of highly expressed unsaturated fatty acids on the cell membrane in the presence of lipoxygenase (Stockwell et al., 2017; Ursini and Maiorino, 2020). Distinct from other types of cell death, ferroptosis results in mitochondrial shrinkage with increased membrane density and reduced cristae, while changes in the nucleus are not evident. It also differs from apoptosis, necrosis, and pyroptosis with respect to biochemistry and gene expression (Xie et al., 2016). Accumulating evidence has demonstrated that ferroptosis dysfunction is associated with a variety of diseases. However, any association has not been reported with AS.

In this study, we investigated the biological mechanisms underlying AS pathogenesis using several bioinformatic approaches. Gene expression profiles of patients with AS and healthy populations were downloaded from the gene expression omnibus (GEO) database, and the AS transcriptome data were further processed and analyzed. Differentially expressed genes (DEGs) were identified between the treatment and control groups, and LASSO regression analysis was used to construct predictive models based on ferroptosis-related genes (FRGs). Single sample gene enrichment analysis (ssGSEA) was performed to assess the level of ferroptosis in each sample, and samples were screened for biomarkers or therapeutic targets *via* weighted gene co-expression network analysis (WGCNA). A protein-protein interaction (PPI) network was established to elucidate prospective target genes involved in AS. The association between key genes and immune cell infiltration levels was assessed using CIBERSORT, to further investigate the function of key genes in AS, and explore their correlation in immune microenvironment. Finally, gene ontology (GO) and pathway analyses using the Kyoto Encyclopedia of Genes and Genomes (KEGG) were performed to predict the biological functions of the key genes, these studies have attempted to highlight transcriptome differences between various phenotypes and disease. which demonstrated the potential link between ferroptosis and AS. This study may provide novel insights for developing strategies for AS treatment and assessing potential molecular targets.

## Materials and methods

### Data acquisition and pre-processing

Gene expression profile data for patients with AS were obtained from datasets GSE 25101 (Pimentel-Santos et al., 2011) (https://www.ncbi.nlm.nih.gov/geo/query/acc.cgi?acc=GSE25101) and GSE41038 (https://www.ncbi.nlm.nih.gov/geo/query/acc.cgi?acc=GSE41038) in the GEO database (Barrett et al., 2007). Dataset GSE25101 (GPL6947 Illumina HumanHT-12 V3.0 expression beadchip) comprised the data of 32 samples, among which 16 were AS samples, while the other 16 were normal controls. Dataset GSE73754 (GPL6883 Illumina HumanRef-8 v3.0 expression beadchip) comprised data corresponding to 72 samples in total, with fifty-two AS samples and twenty normal control samples. We normalized and merged the downloaded expression matrices using the normalizeBetweenArrays function in the limma package of R (Bolstad et al., 2003). Ferroptosis genes were obtained from the FerrDb database (http://www.zhounan.org/ferrdb) (Zhou and Bao, 2020). The FerrDb database comprises 784 articles on ferroptosis downloaded from the PubMed database and the information extracted from the papers on regulatory factors and biomarkers of ferroptosis and corresponding diseases.

### Development of predictive model

Differentially expressed FRGs in patients with AS were used to construct predictive models. LASSO regression analysis was performed on the training cohort using the Glmnet package in R (Friedman et al., 2010). The LASSO algorithm could reduce the dimensionality of high-dimensional data and depict the features of the data with a model comprising fewer variables (Gui and Li, 2005). Ten-fold cross-validation was used to avoid overfitting the model constructed from the training cohort. Finally, a scoring system was established based on the regression coefficients calculated from the LASSO regression analysis. All patients with AS were divided into high-risk and low-risk groups based on the threshold values derived from the package “survival” in R. A univariate COX regression and a univariate COX regression analysis were performed to screen RNA modifier genes that play significant roles, and results were visualized using the package “forestplot” in R.

### Characteristic gene enrichment analysis

GO functional annotation analysis is a common method for conducting large-scale gene functional enrichment analysis, which includes functions such as biological process (BP), molecular function (MF), and cellular component (CC). GO functional annotation analysis of the signature genes was performed using the package ClusterProfiler in R (Yu et al., 2012), and the significant GO results were further visualized using the package treemap in R. A *p*-value < 0.05 was considered statistically significant in this study.

### Single sample gene enrichment analysis

The principle of ssGSEA is similar to that of GSEA. The aforementioned method is an implementation proposed mainly for single samples that cannot be analyzed with GSEA. Five FRG sets were obtained from the FerrDb database (Zhou and Bao, 2020) (http://www.zhounan.org/ferrdb/index.html), and the level of ferroptosis in each sample was assessed based on the expression levels of ferroptosis-specific marker genes. ssGSEA analysis was performed using the “GSVA” package in R.

### Weighted gene Co-expression network analysis

WGCNA is a statistical method used in systems biology to describe the patterns of gene association networks between different samples. It can also be used to identify highly synergistic sets of genes and candidate biomarkers or therapeutic targets based on the endogeneity of these gene sets and the association between the gene sets and corresponding phenotypes. In contrast to focusing solely on DEGs, in WGCNA, data of thousands of the most highly variable genes or complete genes are utilized to identify gene sets of interest, and their association with phenotypes is examined using significant association analysis (Langfelder and Horvath, 2008). We used the WGCNA package in R (Langfelder and Horvath, 2008) to establish a weighted gene correlation network and identify the clustering modules based on clinicopathological features and ferroptosis gene sets (Botía et al., 2017). The correlation among module eigengenes, clinicopathological features, and ferroptosis gene sets was evaluated to identify highly correlated modules. Gene significance (GS) > 0.5 and module membership (MM) > 0.7 were used as the screening parameters.

### Construction of the protein-protein interaction network

PPI networks are composed of interactions between different individual proteins. They are involved in various aspects of physiological processes, such as biological signaling pathways, regulation of gene expression, energy and matter metabolism, and cell cycle regulation. Systematic analysis of the interactions between a large number of proteins is important for understanding how proteins co-function in biological systems. It is also essential for understanding the mechanisms underlying biological signaling and energy and matter metabolism under specific physiological conditions, such as diseases. Moreover, it helps to understand the functional connections among proteins. Spearman correlation analysis was conducted between characteristic FRGs and screened module genes with the screening conditions of cor >0.4 and *p* < 001. The ferroptosis-related AS characteristic gene-module gene network was visualized using Cytoscape (version: 3.9.0). The gene with the highest node degree in the network was selected as the hub gene for this study.

### Gene set enrichment analysis of hub genes

GSEA is used to determine the contribution of genes in a predefined gene set to a particular phenotype by studying their distribution in a table of genes arranged in the order of phenotypic correlation (Subramanian et al., 2005). Two gene sets, “c2.kegg.v7.4.symbols” and “c5.go.v7.4.symbols”, were obtained from the MSigDB database. GSEA of hub genes was performed with both gene sets separately. GO analysis based on GSEA was performed using the “clusterprofiler” package in R [7]. A *p*-value < 0.05 was considered statistically significant (Liberzon et al., 2015).

### Key genes and immune cell infiltration analysis

CIBERSORT is an algorithm for deconvolution of expression matrices of immune cell subtypes based on linear support vector regression. CIBERSORT was originally used for the analysis of tumor microenvironment (TME) and is now increasingly being employed in the characterization of ICI in non-tumor tissues (Ge et al., 2021). Immune cell infiltration (ICI) analysis of AS can provide important guidance for AS research and help predict prognosis after treatment. RNA-Seq data were used to estimate cellular infiltration in the AS and control groups (Newman et al., 2019). ICI analysis was performed for both AS and control samples using the CIBERSORT algorithm. Pearson correlation coefficients between the expression of key genes and immune cell infiltration levels were calculated to assess the relationship between key genes and ICI.

### Identification of characteristic immune cell subtypes and hub gene validation

Based on the results of CIBERSORT, “ConsensusClusterPlus “package in R (Wilkerson and Hayes, 2010) (http://www.bioconductor.org/packages/release/bioc/html/ConsensusClusterPlus.html) was used for clustering analysis of AS samples, with repetitions set to 50 (reps = 50) and a resampling rate of 80% (pItem = 0.8). Samples were classified into different groups based on the number of immune cells present in each sample. To validate these groupings, principal component analysis (PCA) was performed to examine the expression of all genes, and results were visualized with the “ggplot2” package in R. The expression of hub genes among different groups was also evaluated.

## Results

### Overall flowchart of experimental design

**FIGURE 1:**
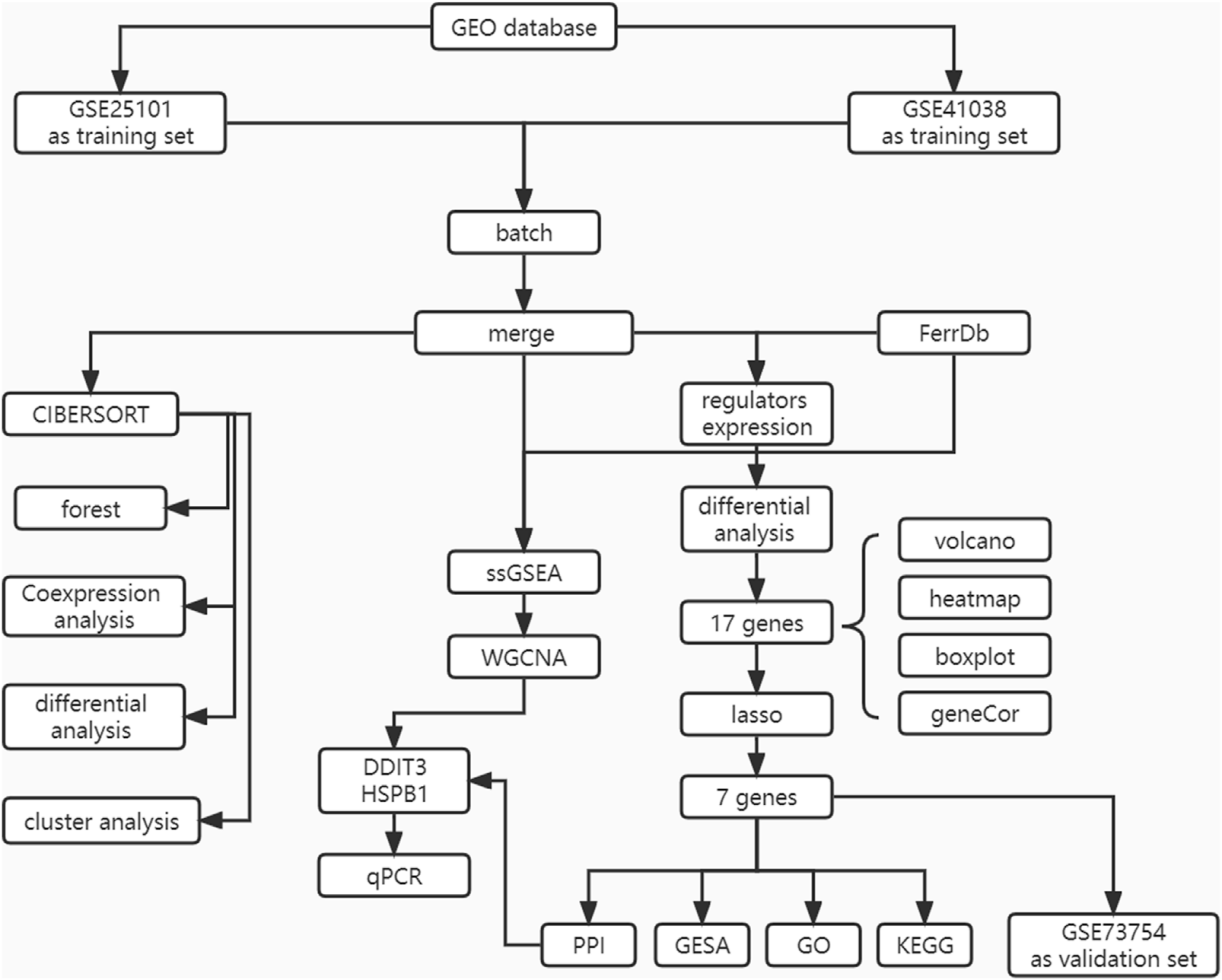
The flow chart of the current study. This study compared the expression characteristics of FRGs in the AS tissue and in the normal tissues, then constructed a prediction model and a FRGs interaction network, and performed molecular subtype analysis to screen out the diagnosis landmark of AS.

The flowchart of the study design is shown in Figure 1. First, the expression data corresponding to patients with AS and healthy patients were downloaded from the GEO database. The samples were then identified and subjected to ICI phenotyping and correlative functional analysis using CIBERSORT. Characteristic genes were selected as diagnostic markers using LASSO regression and validated in the database GEO73754.WGCNA was applied to identify co-expressive gene modules in AS, explore the association between the gene networks and phenotypes of interest, and discover core genes in the network. Finally, the genes that were identified as both core genes and diagnostic markers were considered as key genes, and the correlation between key genes and molecular subtypes and immune cells was determined.

### Differentially expressed genes in AS and normal samples

**FIGURE 2:**
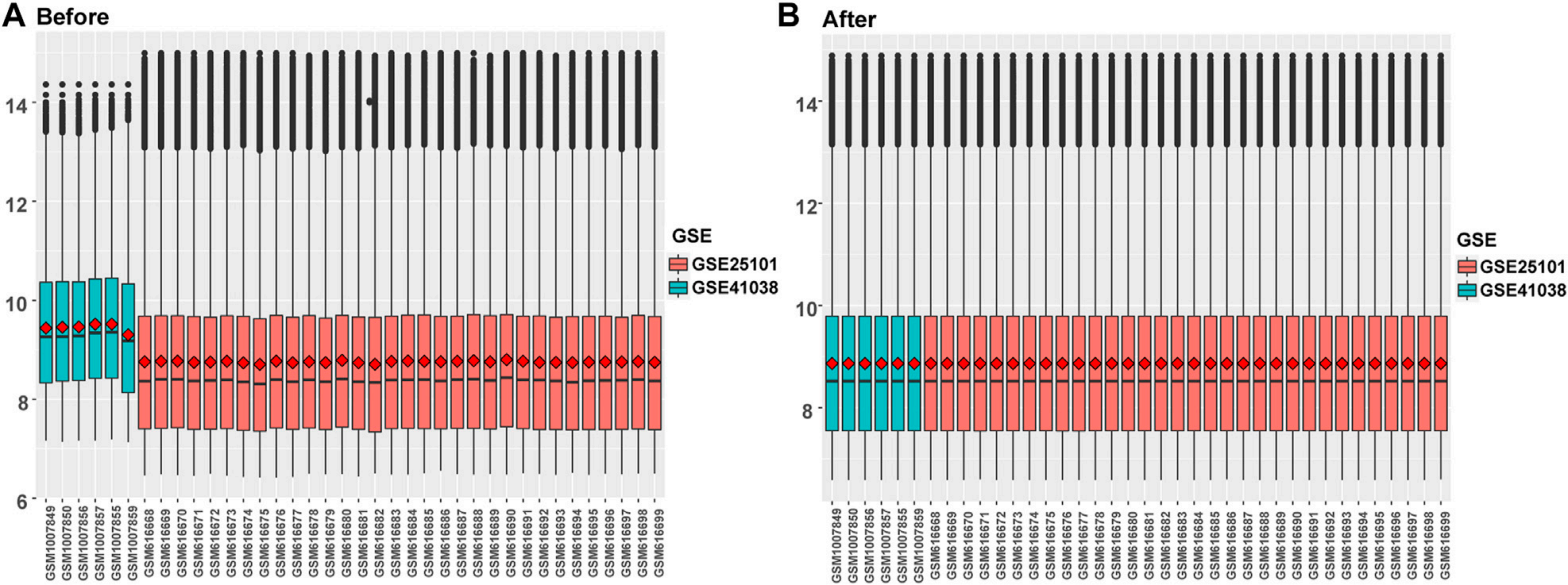
Data integration of typhoon expression matrix. **(A)**. The overall gene expression values of the two GEO data sets before correction, **(B)** The overall gene expression values of the two GEO data sets after correction.

**FIGURE 3:**
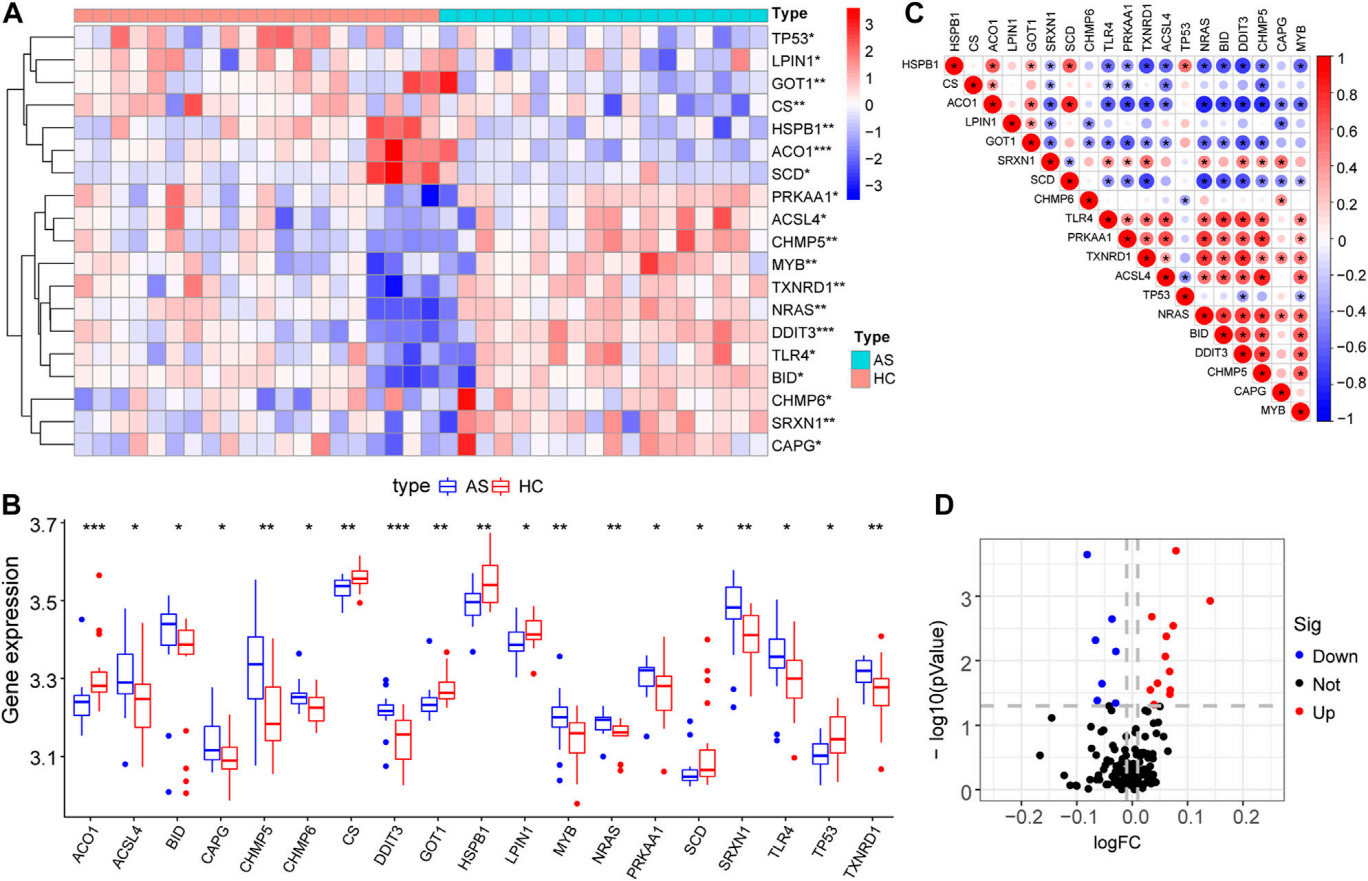
Expression characteristics of FRGs. **(A)** Heat map shows the expression characteristics of FRGs in the AS tissue compared with those in the normal tissues. Red stands for high expression level, and blue for low expression level; **(B)** Box plot shows the difference in the expression of FRGs in the AS tissue and normal tissues in the fifty-two AS samples and twenty normal control samples, with significant DEGs. **(C)** Correlation analysis of FRGs, positive correlation is represented by color red while negative correlation is represented by color blue. **(D)** Volcano plot shows the nineteen differentially expressed FRGs that were identified in the AS tissue compared with those in the normal tissues.

Differential gene expression analysis was firstly performed on integrated data (Figures 2A,B) using the limma package to evaluate the effect of gene expression levels in the AS tissue compared to those in normal tissues. Nineteen differentially expressed FRGs were identified in the AS tissue compared with those in the normal tissues (Figures 3A, B, D) including 12 genes with upregulated expression and seven genes with downregulated expression. The following genes showed upregulated expression: ACSL4, BID, CAPG, CHMP5, CHMP6, DDIT3, MYB, NRAS, PRKAA1, SRXN1, TLR4, and TXNRD1, while the following genes showed downregulated expression: ACO1, CS, GOT1, HSPB1, LPIN1, SCD, and TP53. Co-expression analysis showed that the expression of TLR4 was significantly positively correlated with the expression of most DEGs, including PRKAA1, TXNRD1, ACSL4, NRAS, BID, DDIT3, CHMP5, and MYB (Figure 3C).

### Construction of AS prediction model and gene screening

**FIGURE 4:**
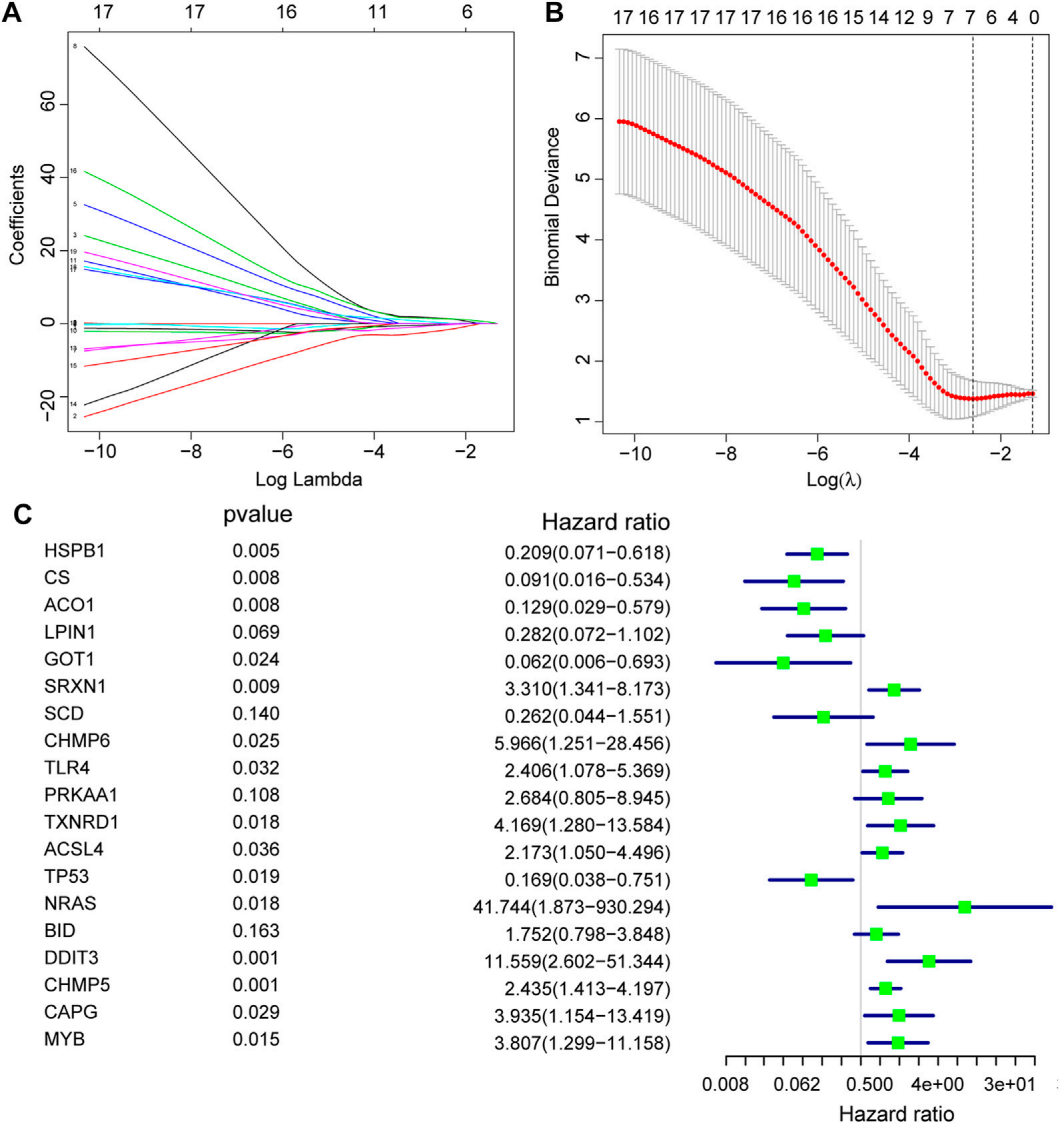
Construction of FRGs prediction model. **(A,B)**: Determine the best penalty value in the LASSO regression algorithm, and screen the hub genes most related to the AS. **(C)**: Forest plots were used to display the screened RNA modifiers.

**FIGURE 5:**
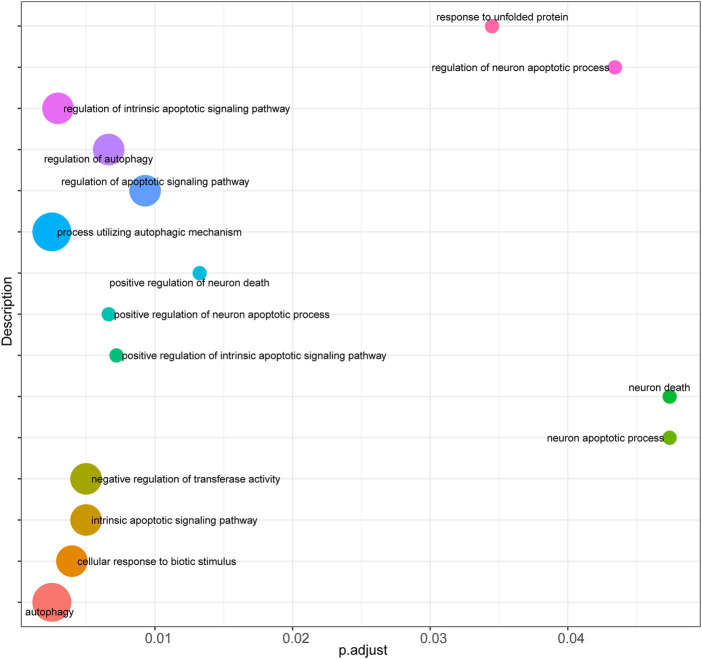
Functional enrichment analysis of key genes. Bubble plot was performed to display functional enrichment analysis of the selected key genes.

Firstly, prediction models for AS were constructed based on the expression levels of FRGs using LASSO regression (Figures 4A,B), and the resultant prediction models depicted the expression of seven genes. Secondly, COX regression was used to screen the genes associated with AS (Figure 4C). Furthermore, functional enrichment analysis was performed to examine the selected key genes, which showed that these genes were mainly enriched in GO terms such as response to unfolded protein, regulation of neuron apoptotic process, regulation of intrinsic apoptotic signaling pathway, cellular response to biotic stimulus, negative regulation of transferase activity, intrinsic apoptotic signaling pathway, regulation of autophagy, regulation of apoptotic signaling pathway, positive regulation of neuron apoptotic process, and positive regulation of intrinsic apoptotic signaling pathway (Figure 5).

### Weighted gene Co-expression network and module enrichment analyses

**FIGURE 6:**
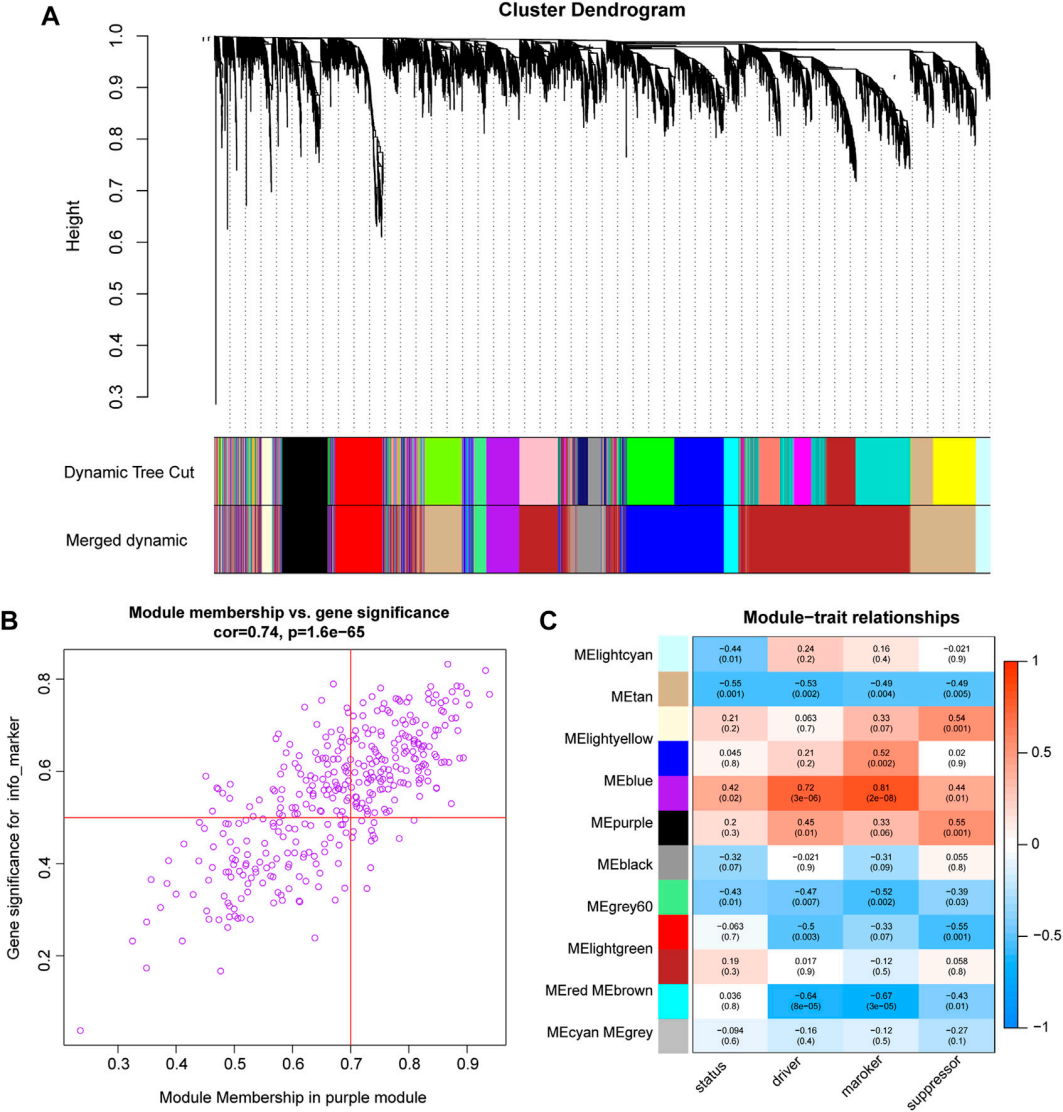
WGCNA analysis **(A)**. Cluster dendrogram of co-expression modules evaluated the correlation of each color module with ferroptosis **(B)**. The purple module was significantly positively correlated with each indicator of ferroptosis. The marker function demonstrated the strongest correlation. **(C)**. scatter plot shows the 104 genes in the purple module of the marker function.

WGCNA was performed on the expression profiles of the combined GEO dataset, and the correlation of each color module with ferroptosis was evaluated (Figure 6A). The purple module was significantly positively correlated with each indicator of ferroptosis, with the marker function demonstrating the strongest correlation (R = 0.81, P = 2e-08, Figure 6B). Therefore, the study was conducted with the purple module of the marker function, which was selected as the subject. In this module, genes (n = 104) with GS > 0.5 and MM > 0.7 were selected for enrichment analysis (Figure 6C).

### Screening key genes and single sample gene enrichment analysis

**FIGURE 7:**
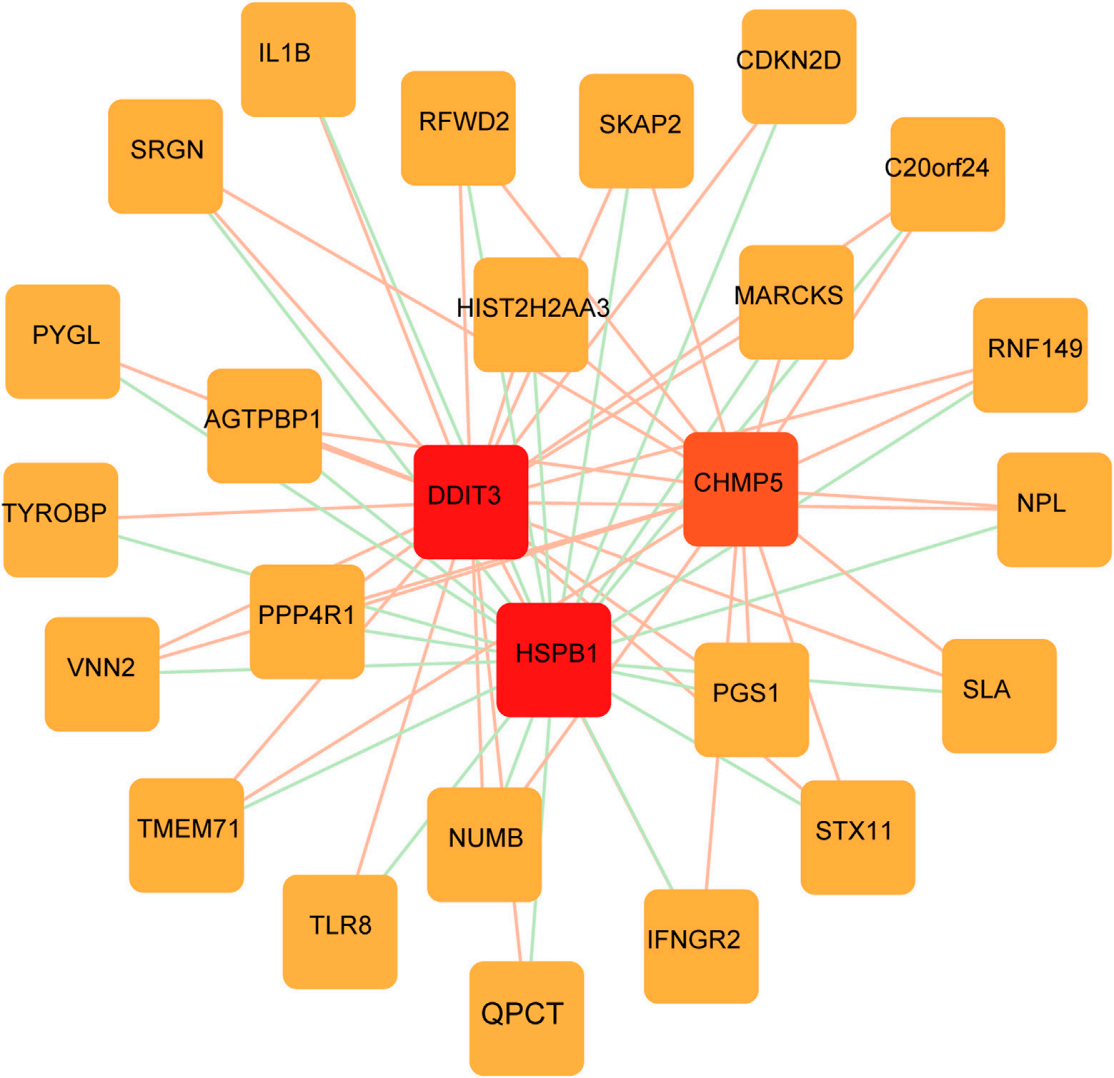
FRGs network construction. PPI network shows the genes identified as important RNA modifiers including *DDIT3* and *HSPB1*.

A PPI network of FRGs was constructed to further screen key FRGs (Figure 7). Genes such as DDIT3 and HSPB1 were identified as important RNA modifiers according to the constructed network.

**FIGURE 8:**
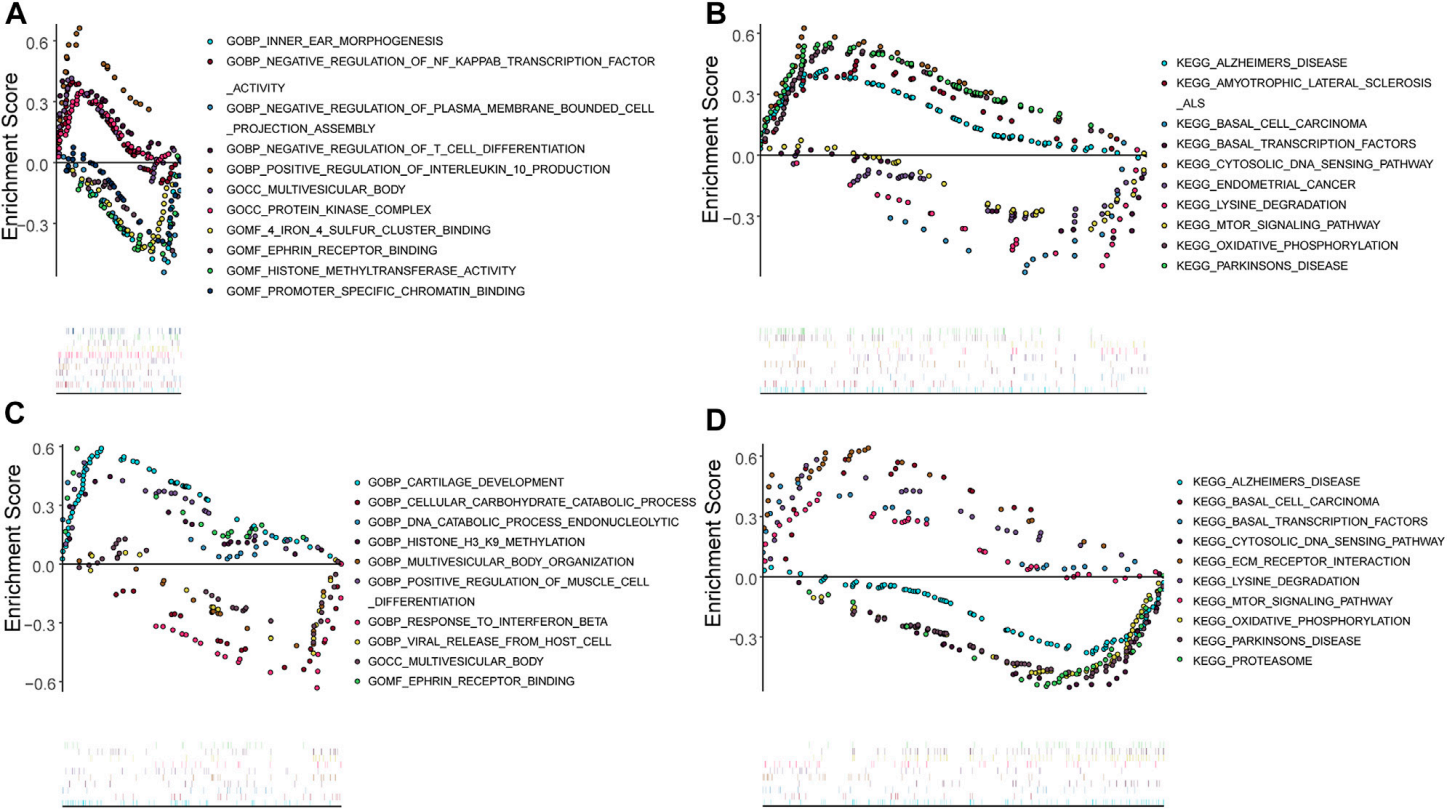
Single gene GSEA analysis **(A,B)**: Single gene GSEA-GO and GSEA-KEGG analysis of DDIT3; **(C,D)**: Single gene GSEA-GO and GSEA-KEGG analysis of HSPB1.

The potential functions of key genes were assessed using ssGSEA (Figure 8). *DDIT3* was primarily enriched in pathways related to amyotrophic lateral sclerosis, basal transcription factors, cytosolic DNA sensing, lysine degradation, mTOR signaling, oxidative phosphorylation, and Parkinson’s disease (Figures 8A,B), while *HSPB1* was mainly enriched in cytosolic DNA sensing pathway, ECM receptor interaction, lysine degradation, the mTOR signaling pathway, oxidative phosphorylation, proteasome-related pathways, and pathways for Alzheimer’s and Parkinson’s disease (Figures 8C,D).

### Correlation analysis of immune cell infiltration and key genes

**FIGURE 9:**
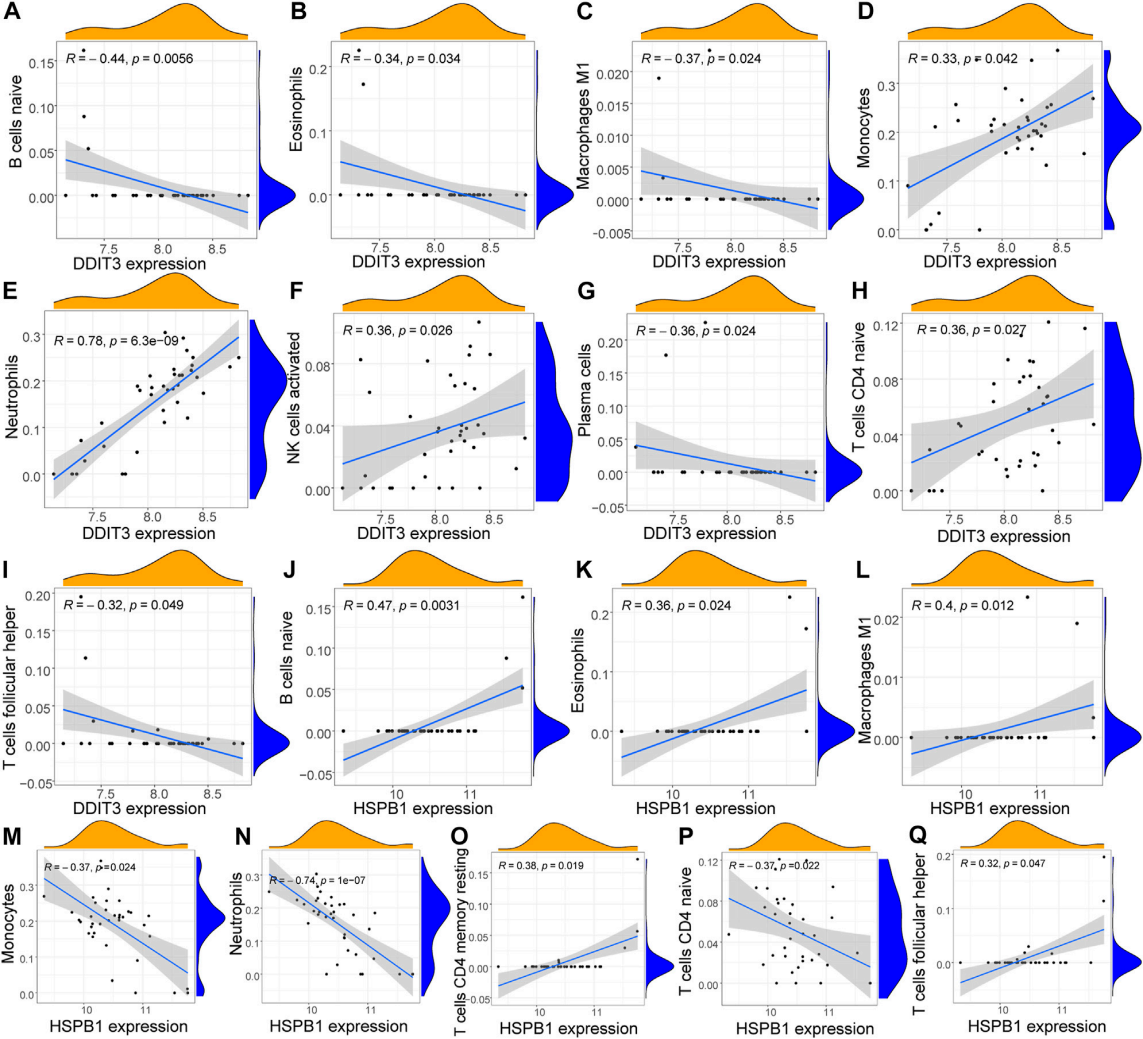
Correlation analysis of key genes and immune cells **(A–I)**: Correlation analysis of DDIT3 and immune cells, the slope is the magnitude of correlation, and the *p*-value represents the significance level. Cells with *p* < 0.05 and correlation coefficient >0.3 were screened for display; **(J–Q)**: Correlation analysis of HSPB1 and immune cells the slope is the magnitude of correlation, and the *p*-value represents the significance level. Cells with *p* < 0.05 and correlation coefficient >0.3 were screened for display.

To further investigate the function of key genes in AS, correlation was analyzed between key genes and the immune microenvironment. The results of the correlation of CIBERSORT results with hub gene expression levels indicated that the expression of DDIT3 was significantly positively correlated with the infiltration levels of many innate and acquired immune cells, including naive B cells, eosinophils, M1 macrophages, monocytes, neutrophils, activated NK cells, plasma cells, naive CD4 T cells, and T follicular helper cells. Here, the correlation with neutrophils was the strongest (R = 0.78) (Figure 9). The expression of HSPB1 was significantly correlated with the infiltration levels of many innate and acquired immune cells, including naive B cells, eosinophils, M1 macrophages, monocytes, neutrophils, resting memory CD4 T cells, naive CD4 T cells, and T follicular helper cells. The strongest negative correlation was observed with neutrophils (R = -0.74) (Figure 9).

### Ankylosing spondylitis immuno-molecular typing

**FIGURE 10:**
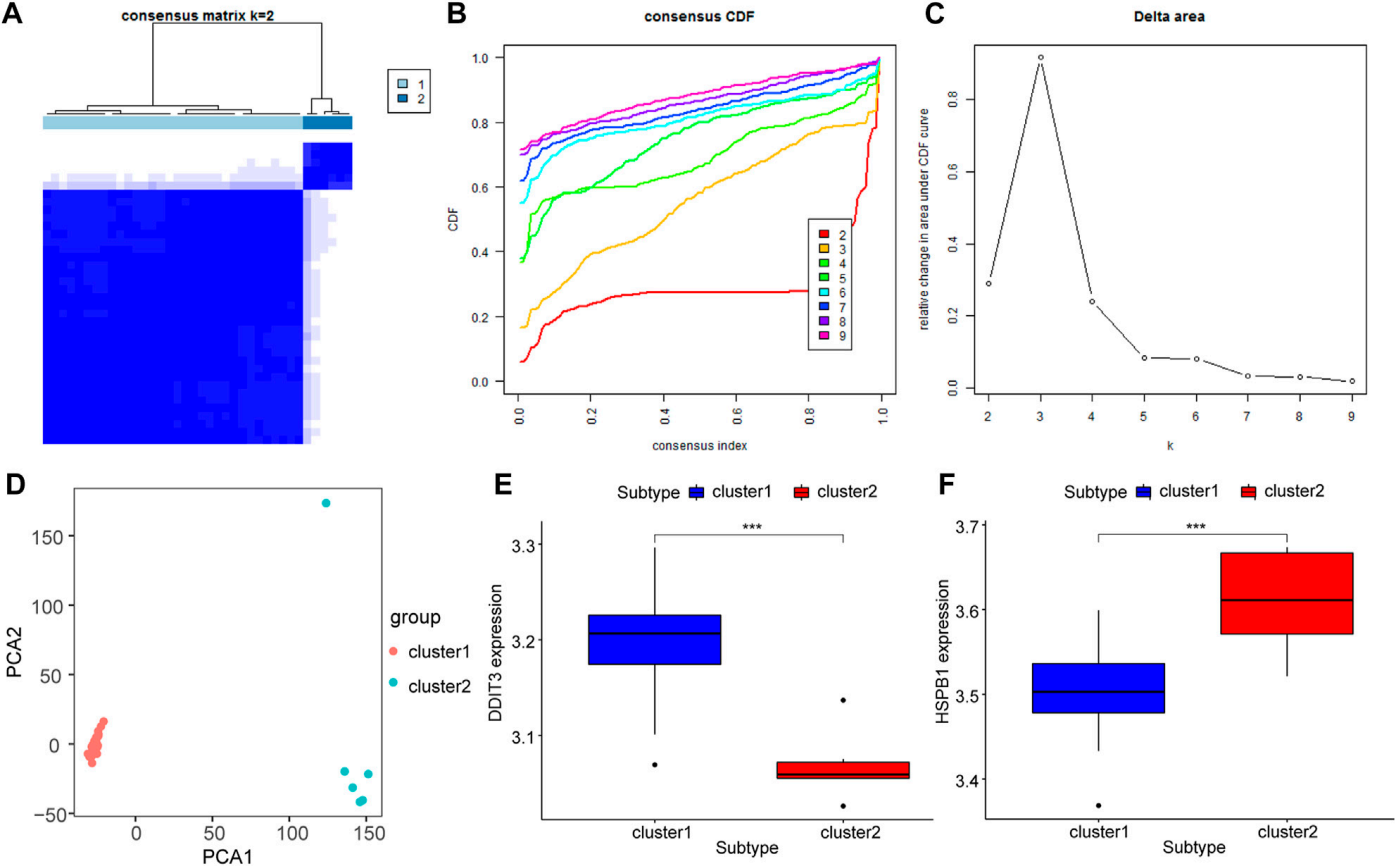
Molecular Types of Ankylosing Spondylitis. **(A–C)**: Clustering of synovial samples based on differential genes for FRGs. **(D)** PCA analysis of different groups, red is cluster A and green is cluster **(B)**; **(E,F)**: The differentially expressed key genes of DDIT3 and HSPB1 in different groups, blue is Cluster A, red is Cluster **(B)**.

Unsupervised consensus clustering (UCC) was applied to all samples based on their immune cell contents to further explore the biological characteristics of gene expression in AS tissues. All samples were divided into two subtypes (subtype 1: n = 32; subtype 2: n = 6, Figures 10A–C), and the results of PCA indicated high separation quality (Figure 10D). Further differential expression analysis of the hub genes in group 1 and group 2 showed that DDIT3 and HSPB1 were significantly differentially expressed in different subgroups (*p* < 0.001) (Figures 10E,F). Accordingly, we speculated that DDIT3 and HSPB1 could be used as key genes for AS.

## Discussion

Ferroptosis is a newly discovered type of regulated cell death (RCD), characterized by iron-dependent lipid peroxidation (Ursini and Maiorino, 2020). Since the introduction of the concept of ferroptosis, countless studies have focused on its role in various diseases. Many studies have particularly demonstrated the relationship between ferroptosis and the treatment or prognosis of various cancers (Ye et al., 2020a; Chen et al., 2021) Recently, Rong et al. (Rong et al., 2022) has demonstrated that ferroptosis may be implicated in AS with potential molecular regulation pathways. Although the specific pathways were not revealed previously, it helps researchers with support of subsequent investigation fields. Encouragingly, we further managed to identify diagnostic biomarkers of ferroptosis in AS by multiple datasets and to elucidate its clinical feasibility by conducting clinical experimental validation. However, the association between ferroptosis and AS has not yet been reported. Advances in high-throughput technologies in the last few years have led to remarkable progress in identifying novel AS-associated genes, including genes that encode cytokine receptors, transcription factors, and signaling molecules (Ranganathan et al., 2017). The pathogenesis of AS is complex. Therefore, in this study, we employed several bioinformatic and statistical approaches to explore the underlying molecular and signaling mechanisms of AS progression. We searched for FRGs that may play an important role in AS and their associated biological functions by constructing predictive models of AS and FRGs. We also performed a functional enrichment analysis of the selected key genes. The results of GO-GSEA showed that the biological processes enriched by DEGs may be significantly associated with processes such as cell death and biotic stimulus responses. A total of 19 DEGs associated with ferroptosis, including 12 genes with upregulated expression and seven genes with downregulated expression, were identified with differential expression analysis. We then constructed a predictive model based on FRGs with LASSO regression and screened for genes associated with AS using COX regression. Among the screened genes, several genes have been reported to be associated with AS. For instance, ACSL4 is a protein that plays an important role in fatty acid β-oxidation. The prevalent low body fat levels in patients with AS are a result of high fatty acid oxidation rate. This finding may be attributed to the switch from other metabolic pathways to fatty acid metabolism in the ligaments of patients with AS owing to the important role of insulin signaling (Xu et al., 2015). Another example is TLR4. Enhanced osteogenic differentiation of mesenchymal stem cells (MSCs) is associated with pathological osteogenesis in patients with AS, and TLR4 plays an important role in osteogenesis and AS pathogenesis (Yu et al., 2021). The expression of *HSPB1* and *TLR4* is significantly increased in patients with AS showing persistently severe systemic hyperthermia, which may be associated with activation of the innate immune system (Zauner et al., 2014). Moreover, TP53 plays an important role in the pathogenesis of juvenile ankylosing spondylitis (JAS). For the first time, we combined all these genes to construct our predictive model (Wang et al., 2018).

**FIGURE 11:**
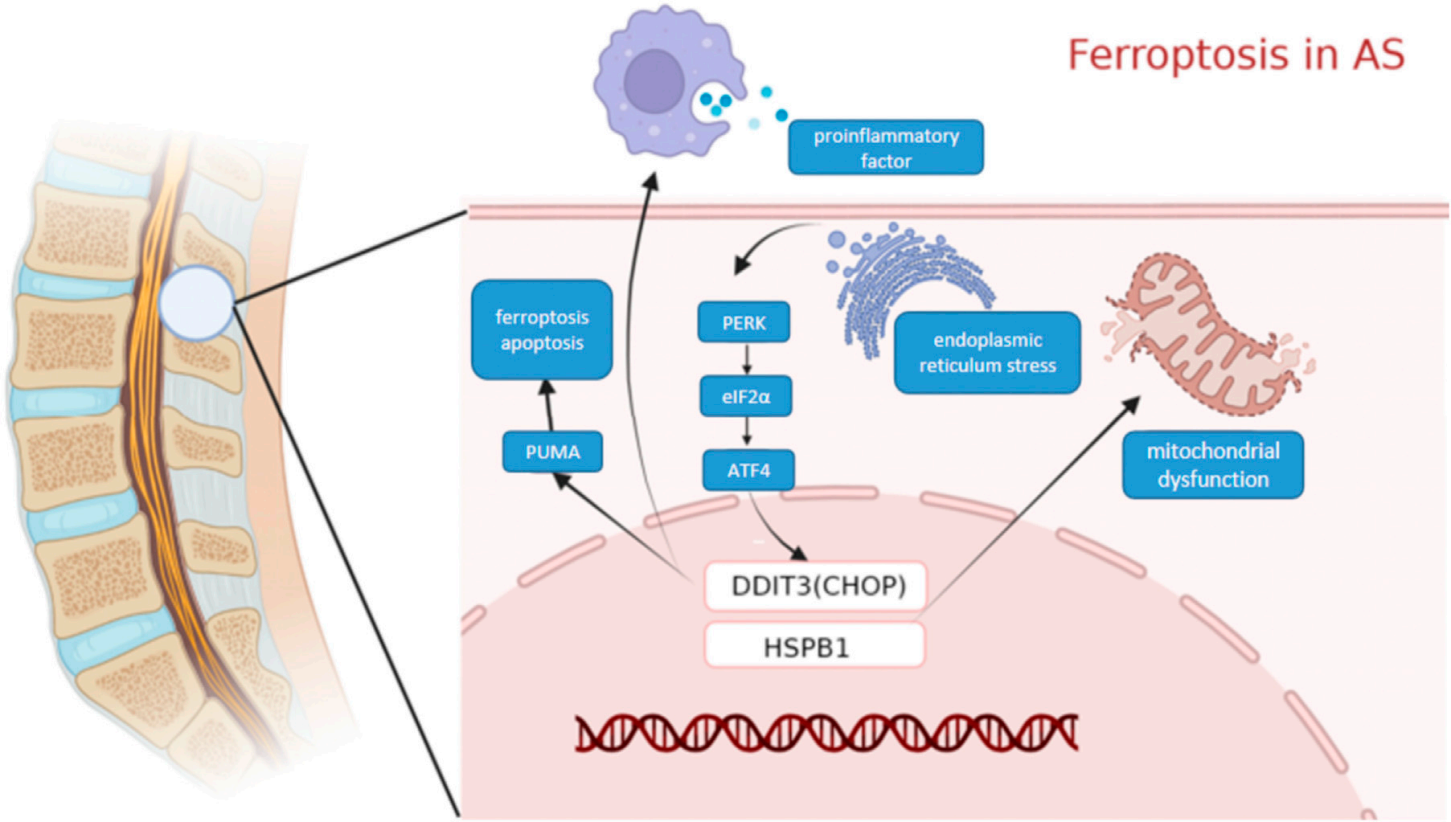
Molecular mechanism diagram. Schematic diagram of the Molecular mechanism ferroptosis in AS.

We used WGCNA to identify clusters of genes expressed in each module in the GEO dataset that were highly associated with ferroptosis. The purple module was significantly positively correlated with each indicator of ferroptosis, yielding a total of 104 genes. A PPI network of ferroptosis was constructed based on the STRING database. DDIT3 and HSPB1(SFigure1), among other genes, were identified as important RNA modifiers in the network. The Schematic diagram of the two key genes DDIT3 and HSPB1 inducing ferroptosis in AS is shown in Figure Figure11.

DDIT3, which encodes C/EBP homologous protein (CHOP), belongs to the CCAAT/enhancer-binding protein (C/EBP) transcription factor family. This family of transcription factors regulates a variety of genes and is involved in various physiological processes such as immune response, cell differentiation, and cell proliferation. DDIT3 can be activated by endoplasmic reticulum (ER) stress and promotes apoptosis, and iron reagents can induce ER stress-mediated activation of the PERK-eIF2α-ATF4-CHOP pathway. The CHOP signaling pathway mediates the expression of PUMA, which is involved in the synergistic interaction between cellular ferroptosis and apoptosis (Lee et al., 2018). Moreover, CHOP can promote the polarization of macrophages and regulate the secretion of pro-inflammatory cytokines. Meanwhile, CHOP overexpression promotes immunosuppression in the tumor microenvironment (Allagnat et al., 2012). In this study, ssGSEA analysis of CHOP revealed that it was mainly associated with pathways and functions involving NF-kB, B cells, and T cells. More than ninety per cent of patients with AS tested positive for HLA-B27, which was mainly expressed on NK cells and CD4^+^ T cells. A significant increase was observed in T cells expressing HLA-B27 receptors in patients with AS, and these T cells were also converted to the Th17 phenotype that was associated with AS pathogenesis (Chan et al., 2005; Bowness et al., 2011). Cytokines produced by NK cells and their cytotoxic effects are involved in the regulation of immune responses and may contribute to the pathogenesis of many immune-mediated diseases, including AS (Kucuksezer et al., 2021). We, therefore, suggest that it is highly possible that it plays an important role in AS.

In addition, HSPB1 belongs to the small heat shock protein (HSP20) family, and it encodes a protein that promotes the proper folding of multiple proteins under environmental stress. A high expression of HSPB1 promotes proliferation and metastasis while protecting the cancer cells from apoptosis (Calderwood and Gong, 2016; Lin et al., 2020). In degenerative tissues, calcification of the vertebral endplate disrupts the internal region of the avascular disc. This would trigger the activation of the stress-sensing HSF-1 complexes, including HSPB1. The severity of IVD rupture was associated with an increased proportion of HSPB1-positive cells (the disc cell population in pathological human discs is associated with increased immunostaining for stress proteins). Examination of HSPB1 using ssGSEA revealed that it was primarily enriched in pathways related to NF-kB, oxidative stress, and interleukin 10. The serum composition of patients with AS was associated with the induction of mitochondrial dysfunction in MSCs and elevation of the intracellular reactive oxygen species (ROS) levels, leading to MSC ageing (Roberts et al., 2006).

To understand the relationship between the expression of key genes and clinical characteristics of AS patients, we applied qRT-PCR to assess the expression of DDIT3 and HSPB1. The results showed that DDIT3 was highly expressed in the tissues of patients with AS, while HSPB1 was less expressed in the tissues of patients with AS. These results suggested that the key genes we selected may be potential targets for the diagnosis and treatment of AS.

Pathological osteogenesis is a major feature of AS. Accumulating evidence demonstrates that inflammation is directly involved in the process of pathological osteogenesis *via* promotion of the production of osteoinductive proteins and the proliferation of osteoprogenitor cells. Here, we analyzed the association between DDIT3 and HSPB1 and the immune response to further explore the characteristics of the inflammatory and immune microenvironments in patients with AS and the possible roles of these key genes. Preliminary analysis suggested that DEGs in AS were enriched in the pathways associated with cellular response to biotic stimuli. Elevated ROS levels have been observed in the leukocytes of patients with AS. These findings suggest that ROS may be a potential mediator of AS pathogenesis. ROS can have various effects on normal cell growth, and small amounts of oxygen radicals can lead to RCD, including apoptosis, autophagy, and ferroptosis (Ye et al., 2020b).

Further immune analysis revealed a higher degree of infiltration of immune cells, such as neutrophils, in AS samples. The innate immune response, wherein neutrophils play an important role, was activated in patients with AS. Polymorphonuclear leukocytes in HLA-B27-positive controls showed increased sensitivity to certain chemoattractants. A large number of such hyperreactive neutrophils accumulating at sites of inflammation may contribute to the inflammatory response in patients with AS (Pease et al., 1989). Correlation analysis of ICI and gene expression performed using CIBERSORT revealed that the two key genes showed a significant positive correlation with the degree of infiltration of various immune cells, such as naive B cells, eosinophils, M1 macrophages, and naive CD4 T cells. DDIT3 and neutrophils had the highest positive correlation coefficient (R = 0.78), while HSPB1 showed the strongest negative correlation (R = -0.74) with neutrophils. Therefore, we propose that the role of key genes in AS may be associated with neutrophil infiltration. The results of self-supervised immune grouping also supported this conclusion.

## Conclusion

In summary, our study found that ferroptosis plays a crucial role in AS. We identified ferroptosis-associated markers in AS diagnosis such as DDIT3 and HSPB1. However, further experiments are required for in-depth study of the mechanism to support our conclusion.

## Data Availability

The datasets presented in this study can be found in online repositories. The names of the repository/repositories and accession number(s) can be found in the article/Supplementary Material.

## Abbreviations

(RCD), regulated cell death; (AS), ankylosing spondylitis; (GEO), gene expression omnibus; (DEGs), Differentially expressed genes; (FRGs), ferroptosis-related genes; (ssGSEA), Single sample gene enrichment analysis; (WGCNA), weighted gene co-expression network analysis; (PPI), Protein-protein interaction; (GO), gene ontology; (KEGG), Kyoto Encyclopedia of Genes and Genomes; (BP), biological process; (MF), molecular function; (CC), cellular component; (ICI), Immune cell infiltration; (TME), tumor microenvironment; (PCA), principal component analysis; (ER), endoplasmic reticulum; (CHOP), C/EBP homologous protein; (ROS), reactive oxygen species; (e.g.), exempli gratia; (MM), module membership; (GS), Gene significance; (MSCs), mesenchymal stem cells; (JAS), juvenile ankylosing spondylitis; (HSP20), heat shock protein.
