# Supplementary material for: Role of ferroptosis-associated genes in ankylosing spondylitis and immune cell infiltration
Source: Front Genet. 2022 Nov 11;13:948290. doi: 10.3389/fgene.2022.948290 (PMC9691995; doi:10.3389/fgene.2022.948290)
Supplement: Supplementary file 1 [file DataSheet1.ZIP › Supplementary materials.docx]

**Supplementary materials**

## Patients

The supraspinous and interspinous ligaments were obtained from patients with AS who underwent osteotomy and patients with common lumbar disc herniation who underwent posterior lumbar interbody fusion (PLIF) in the Department of Orthopaedics, The First Affiliated Hospital of Chongqing Medical University from January 2022 to March 2022. Specimens were excised and transferred immediately to liquid nitrogen. All patients were notified, and all of them provided written informed consent. The study was conducted in accordance with the International Conference on Harmonization’s Good Clinical Practice Guidelines and the Declaration of Helsinki. All patients in the experimental groups were clearly diagnosed with AS according to the axSpA classification criteria of the Assessment of SpondyloArthritis International Society (ASAS) (2009). In contrast, all the patients in the control group were clearly diagnosed with common lumbar disc herniation. Clinical data were obtained from hospital records and pathology reports. The study protocol was approved by the Ethics Committee of the Affiliated Hospital of Chongqing Medical University (Approval Number: 2022-K55).

## Total RNA Extraction

Total RNA was extracted from BC tissues using the UNIQ-10 column total RNA extraction kit (Sangon Biotech). A SMA4000 microspectrophotometer (Merinton Instrument, Inc) and DYY-6C electrophoresis instrument (Liuyi. Beijing) were used to quantify and qualify the extracted total RNA, respectively.

## Reverse Transcription and Quantification with qRT-PCR

The concentrations of total RNA extracted from the supraspinous and interspinous ligaments of patients with AS ranged from 131.81 ng/μl to 1125.56 ng/μl. RNA samples were reverse transcribed using the RR047A cDNA synthesis kit (TaKaRa, China). Quantitative PCR of *CEP55*, *MTFR2*, and *PIMREG* was performed using 2× SG Fast qPCR premix (High Rox, B639273, BBI) and StepOnePlus Real-Time PCR system (ABI, Foster, CA, USA). *GAPDH* was used as an internal control. Primers were designed using Prime 5.0; the sequences are as follows:

GAPDH-F: 5' TGGGTGAACCATGAAGT 3'

GAPDH-R: 5' TGAGTCCTTCCACGATACCAA 3'

DDIT3-F: 5' CCCAGCCACTCCCCATTATCC 3'

DDIT3-R: 5' TTCGGTCAATCAGAGCTCGG 3'

HSPB1-F: 5' GACTGGTACCCGCATAGCC 3'

HSPB1-R: 5' GATTTTGCAGCTTCTGGGCC 3'

## Validation of the Expression of Key Genes using qRT-PCR

The expression of DDIT3 and HSPB1 was assessed using qRT-PCR. The expression of DDIT3 was significantly higher, whereas the expression of HSPB1 was significantly lower in AS samples than in the samples derived from patients with common lumbar disc herniation. It was also found that the expression of DDIT3 in the lower immunity score group were dramatically lower than that in the higher immunity score group.

**Supplementary figure1**

The expression of DDIT3 in the AS samples and common lumbar disc herniation

 The expression of HSPB1 in the AS samples and common lumbar disc herniation
