# Supplementary figures and images for: Role of ferroptosis-associated genes in ankylosing spondylitis and immune cell infiltration
Source: Front Genet. 2022 Nov 11;13:948290. doi: 10.3389/fgene.2022.948290 (PMC9691995; doi:10.3389/fgene.2022.948290)

After

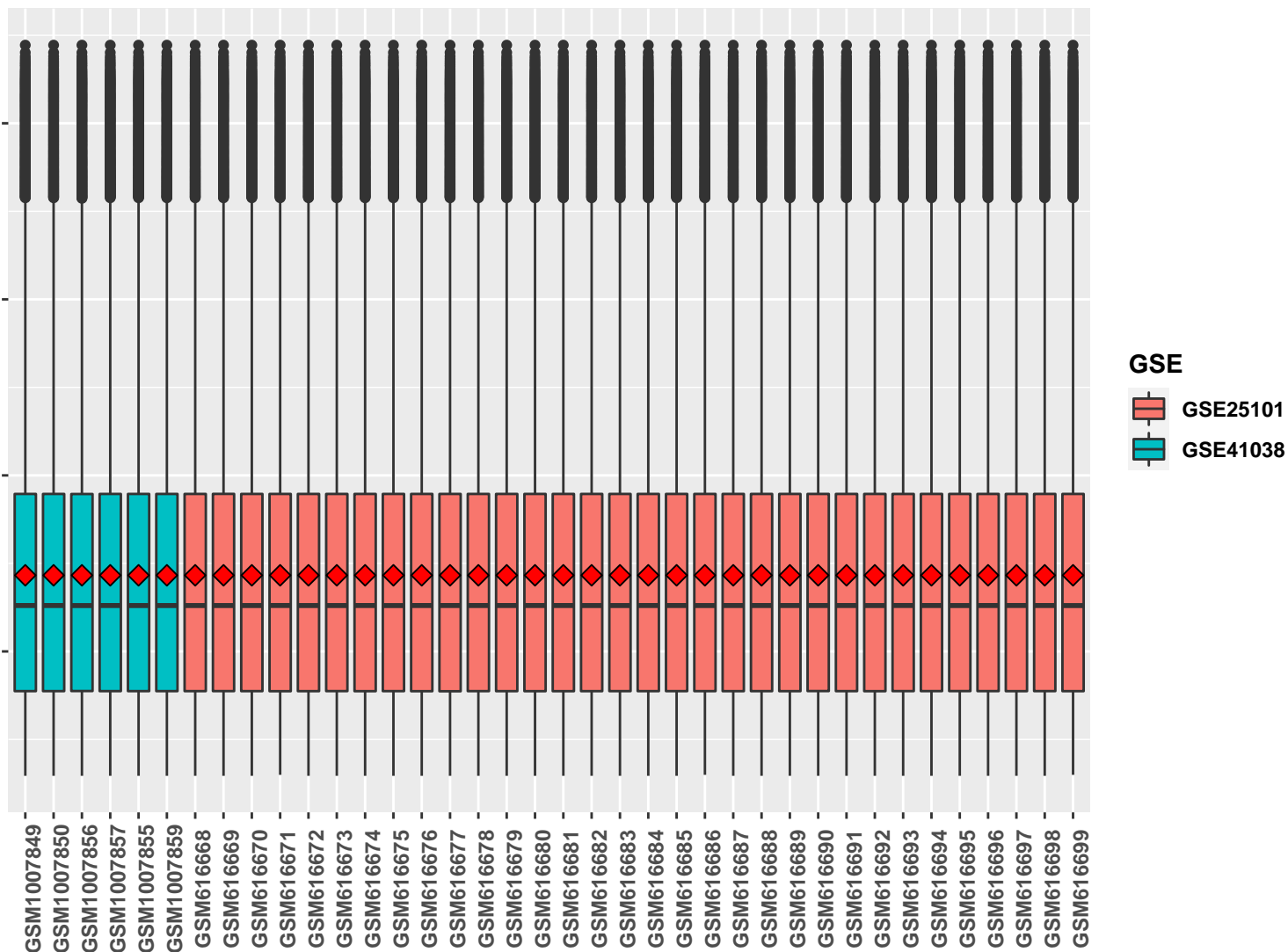

Supplement: Supplementary file 2 [file DataSheet2.ZIP › Supplementary materials of batch correction/After1.pdf]

Before

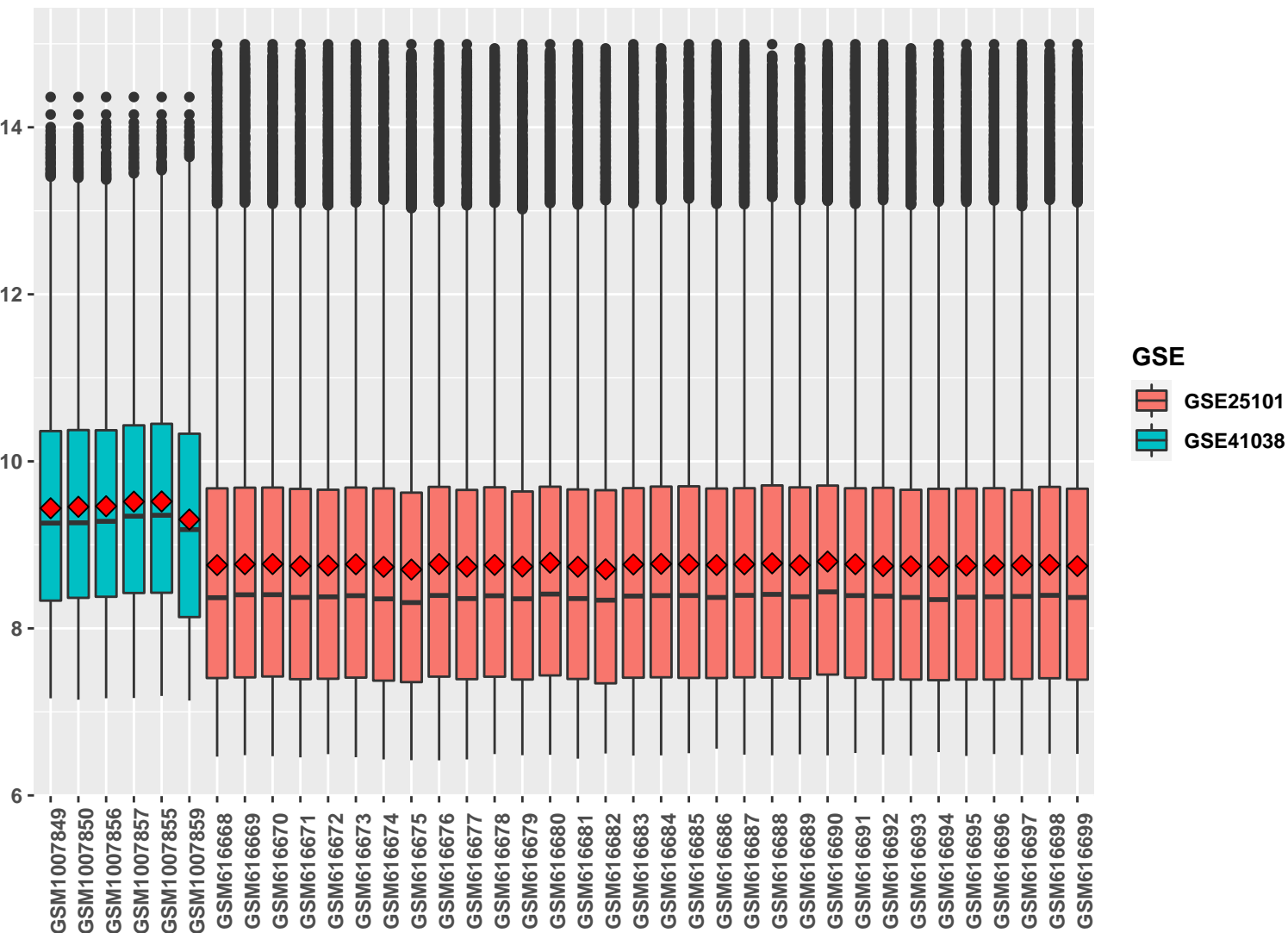

Supplement: Supplementary file 2 [file DataSheet2.ZIP › Supplementary materials of batch correction/Before1.pdf]
